# An innovative approach to three-dimensional ridge augmentation in the posterior mandible: A case report

**DOI:** 10.1016/j.ijscr.2024.110146

**Published:** 2024-08-10

**Authors:** Nadim Sleman

**Affiliations:** Oral and Maxillofacial Surgery, Oral and Maxillofacial Surgery Department, Tishreen University, Latakia, Syria

**Keywords:** Vertical ridge augmentation, Horizontal ridge augmentation, Guided bone regeneration, GBR, Severe bone loss, Bone defects

## Abstract

**Introduction and importance:**

Surgical intervention is necessary to address significant three-dimensional bone loss in the posterior mandibular alveolar ridge when implants are planned, and primary stability cannot be achieved due to anatomical limitations.

The objective of this study is to elucidate the surgical procedures for reconstructing significant bone loss in the posterior mandibular region and to present the outcomes and insights gained from this clinical case.

**Case presentation:**

A 42-year-old woman exhibited first lower molar loss, significant movement of the second molar, and severe bone loss at the same site. Vertical and horizontal bone augmentation was performed to enable the restoration of teeth loss by inserting dental implants.

**Clinical discussion:**

Significant bone loss poses a great limitation in replacing missing teeth, particularly in the posterior mandible, given anatomical constraints. Therefore, it is essential to establish an adequate amount of bone to ensure primary stability for the implants.

**Conclusion:**

This clinical case demonstrates a restoration technique of severe bone loss in the posterior mandible to enable stable dental implant placement, highlighting the importance of combining vertical and horizontal augmentation to overcome anatomical limitations and ensure primary stability.

## Highlights

• Three-dimensional bone augmentation in the severely atrophic posterior mandible is crucial for effective oral rehabilitation.
• Guided bone regeneration is an excellent method for promoting bone formation and ensuring implant stability.
• Implementing innovative surgical techniques enhances bone gain outcomes significantly.

## 1 Introduction

The posterior mandibular alveolar ridge presents unique challenges for implant placement, often requiring surgical intervention to address substantial bone loss. Achieving primary stability, a crucial factor for implant success, is often impossible without bone augmentation. To enhance the longevity and success of dental implant treatment, various bone augmentation procedures are employed. Guided bone regeneration (GBR), specifically for horizontal and vertical ridge augmentation, is widely supported by extensive medical research [[1], [2], [3]], demonstrating high implant survival rates and minimal complications [4,5].

Several GBR techniques have emerged, aiming to achieve predictable and lasting bone regeneration. One promising approach involves utilizing a combination of titanium-reinforced PTFE mesh, autologous bone, and xenograft for vertical bone augmentation. This technique has proven to be reliable and secure, providing a solid foundation for implant placement [6].

Recent clinical and histological studies have shed light on the efficacy of combining anorganic bovine bone-derived mineral (ABBM) with autogenous particulate bone for localized ridge augmentation [3]. This material combination, specifically designed for addressing localized bone defects, appears to promote bone formation, leading to improved implant integration and long-term stability.

The use of the tent screw technique in bone augmentation exhibited positive results in supporting bone grafts [7], indicating a potential benefit in enhancing guided bone regeneration (GBR) when integrated with a titanium-reinforced PTFE membrane.

This case report has been reported in line with the SCARE Criteria [8].

## 2 Case presentation

**Fig. 1:**
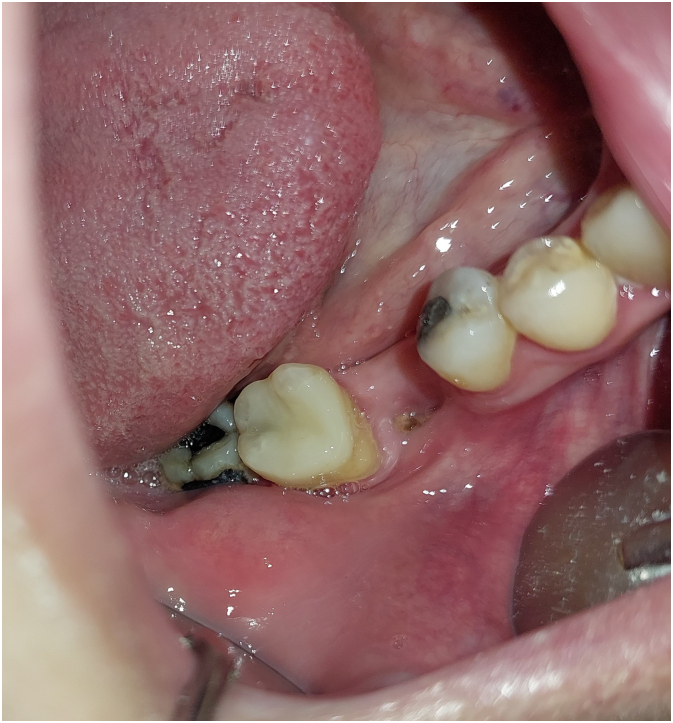
Clinical assessment before surgery.

A healthy 42-year-old woman presented for restoring lost first molar in the posterior region of right mandible. Upon clinical examination, significant bone loss was observed in the extracted first molar region resulting in a pathological defect which requires reconstructive surgery (Fig. 1).

Furthermore, the second molar exhibited a class 2 mobility according to the Miller Classification [9].

**Fig. 2:**
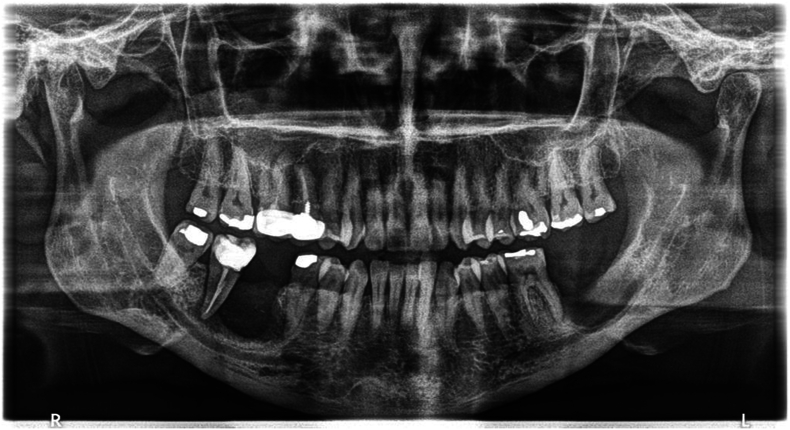
Panoramic x-ray assessment before surgery.

**Fig. 3:**
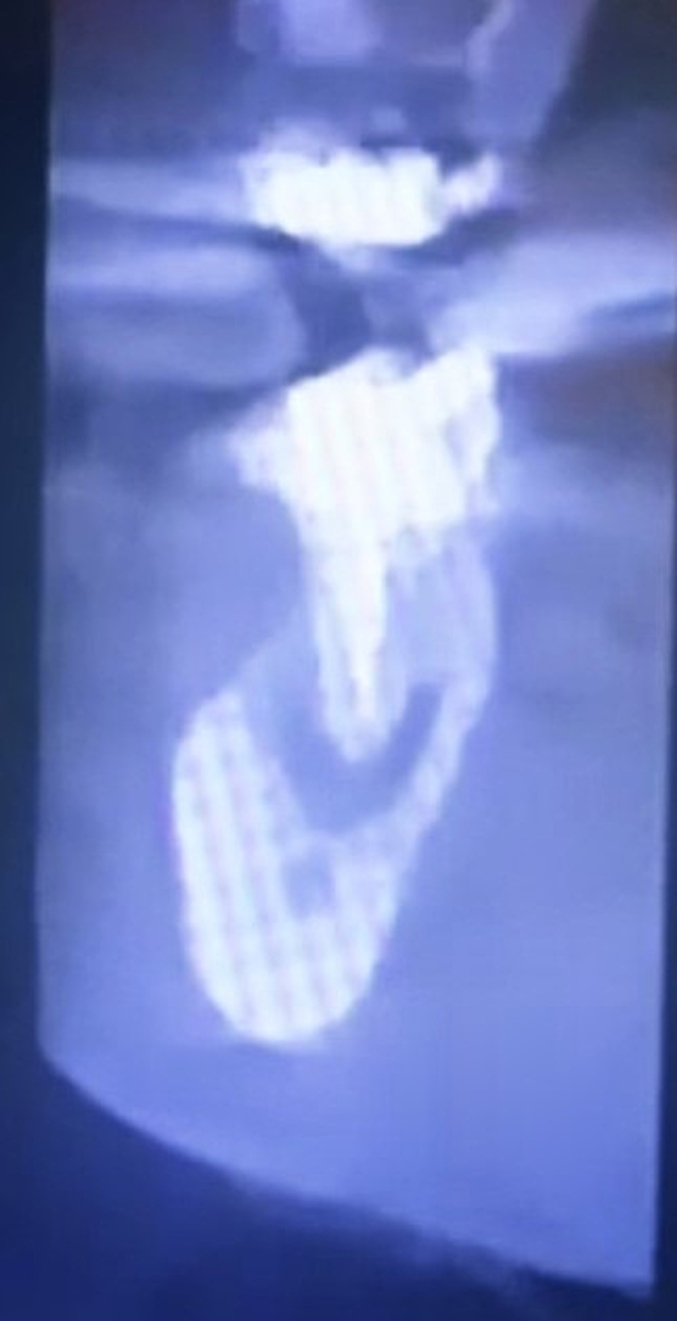
Sagittal view of the first molar site.

**Fig. 4:**
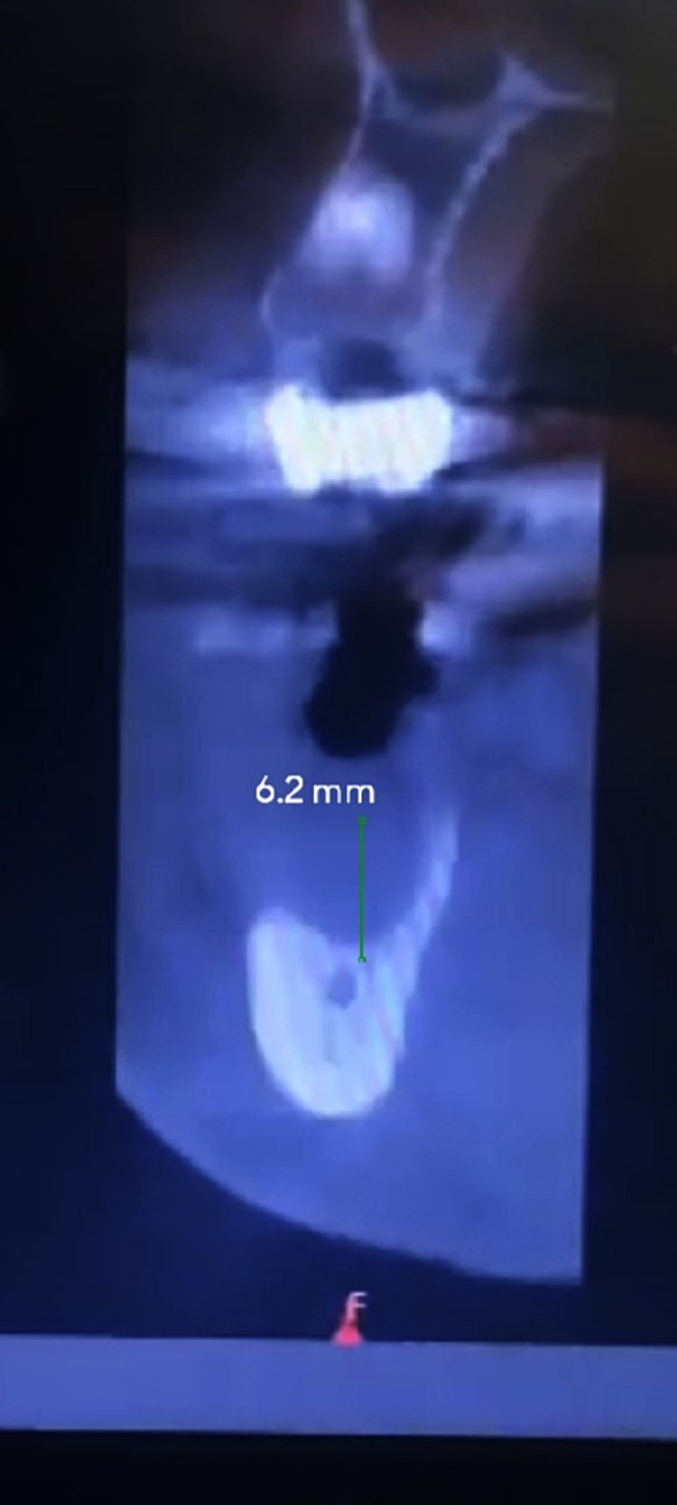
Sagittal view of the second molar site.

Preoperative standardization protocol included clinical and radiological assessment which revealed a significant defect in the alveolar bone extending to the upper border of the inferior alveolar nerve canal (Fig. 2, Fig. 3, Fig. 4).

A cone beam computed tomography (CBCT) scan was performed to guide the planning of the bone augmentation procedure and the subsequent placement of dental implants. As there was insufficient vertical and horizontal bone remaining to achieve primary stability for dental implant placement.

It was determined that extraction of the second molar along with bone augmentation would be necessary to restore the required dimensions of the alveolar bone, allowing for implant insertion at a later stage.

Post-surgery care involved monitoring the patient's recovery and assessing the success of the bone augmentation, providing an opportunity for the rehabilitation of lost teeth through dental implant placement.

## 3 Surgical procedure

The author performed the surgical procedure with the patient under local anesthesia.

The patient was prescribed a 2-g dose of amoxicillin/clavulanic acid (Amoxam®) 875/125 mg before the surgery and continued on antibiotics for 7 days after the procedure.

The patient rinsed out her mouth with a 0.12 % chlorhexidine gluconate mouthwash (Bio fresh-K mouth rinse®). Her Lips and skin were disinfected with povidone‑iodine®.

The surgical site was anesthetized using 2 % lidocaine with 1:200,000 epinephrine.

A trapezoidal full-thickness flap was raised to provide adequate surgical access to the defect in the alveolar bone. Extraction of the second molar was done carefully and the bone was curetted to excise all soft tissue remnants.

**Fig. 5:**
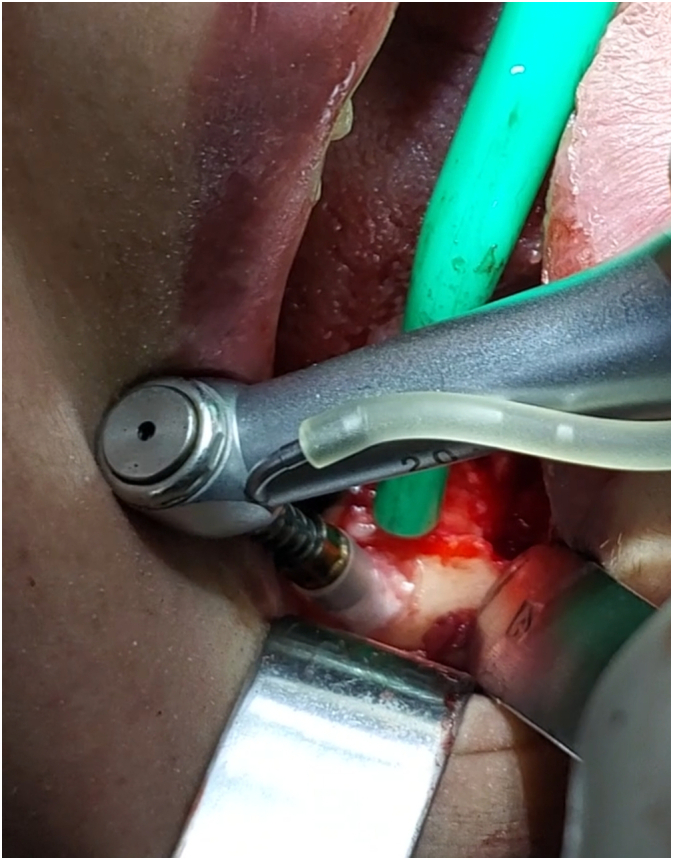
Autogenous bone graft collecting using trephine drills.

**Fig. 6:**
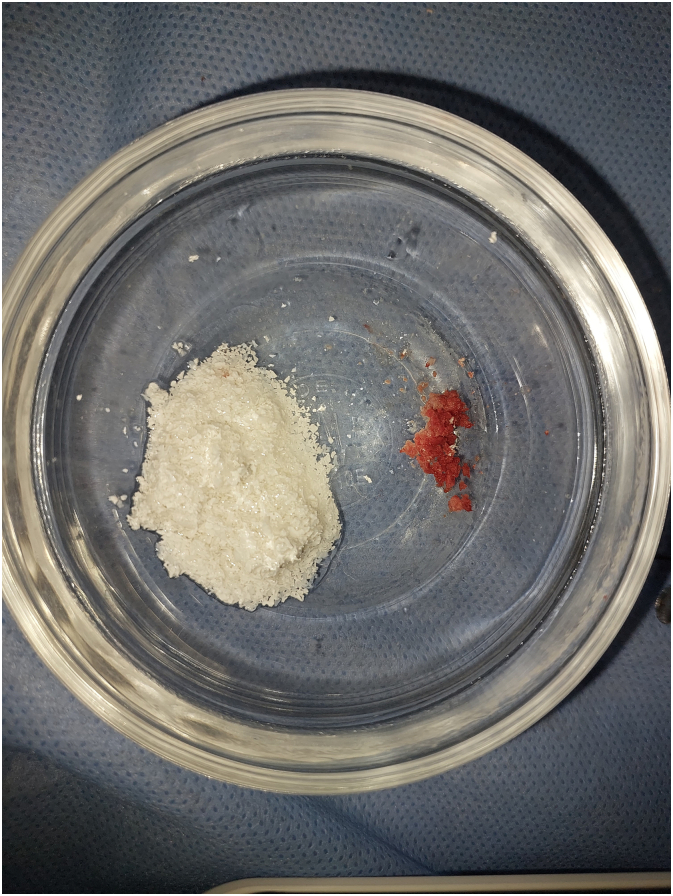
Xenograft and autograft preparation.

An autogenous graft was obtained from the external oblique line using trephine burs (Cowellmedi®) to collect the graft, which was then mixed with 2 g xenograft material (ITP Co., Iran) to enhance healing process (Fig. 5, Fig. 6).

The recipient site was prepared by creating multiple decorticate holes with a round bur to expose the medullary space.

**Fig. 7:**
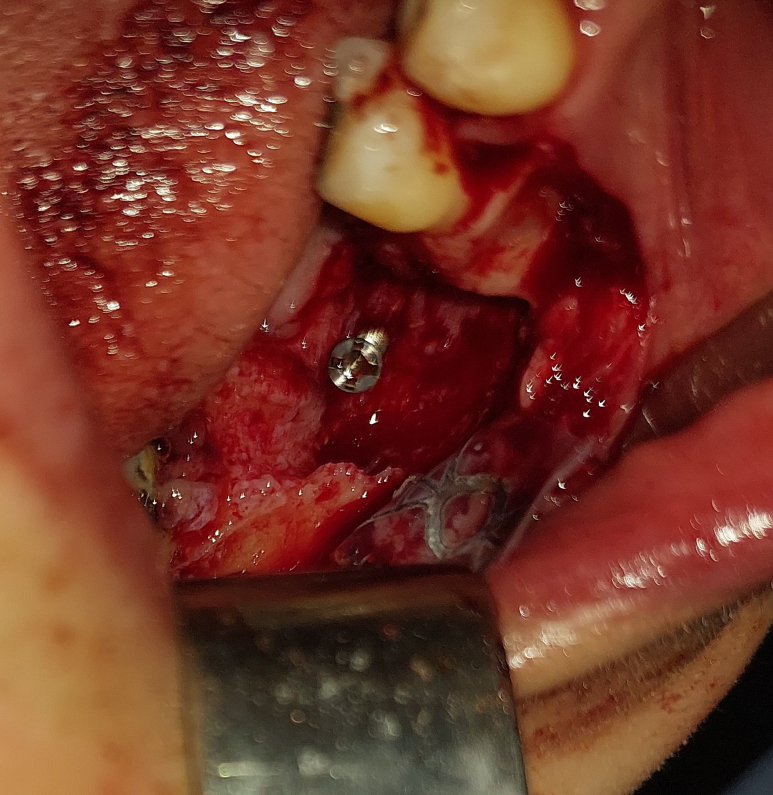
Tent pole technique by inserting the screw 45° buccally.

A tent screw (InnoGenic™ GBR Kit – Cowellmedi) was anchored at the lingual plate and angled buccally to promote the augmented site, which was then shielded by a titanium-reinforced dense polytetrafluoroethylene non-resorbable membrane (InnoGenic™ Wifi-Mesh – Cowellmedi) (Fig. 7).

The ideal placement of the tent screw was determined through CBCT analysis to ensure safe positioning and avoid damage to the inferior alveolar nerve.

**Fig. 8:**
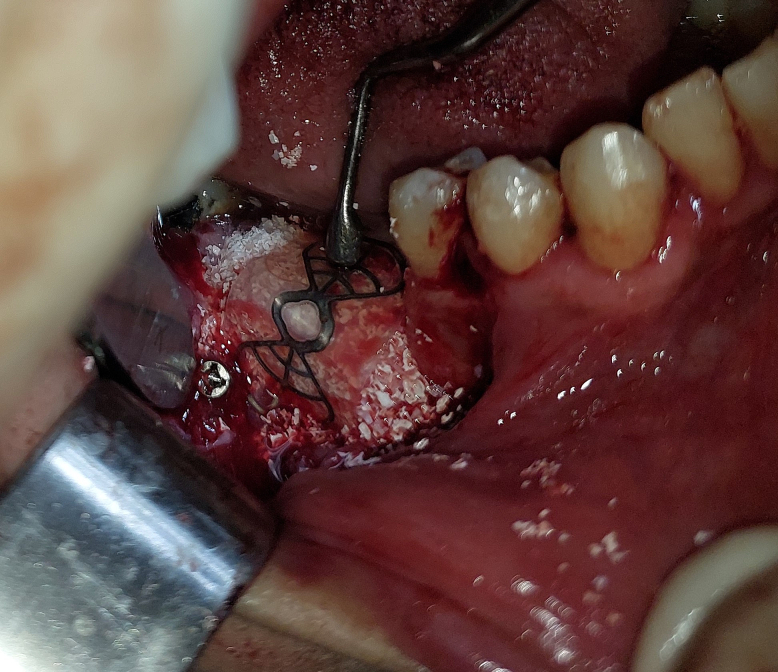
Applying bone graft material and covering it with PTFE membrane.

Bone graft material was applied to the defect and covered with the titanium-reinforced PTFE membrane (Fig. 8).

**Fig. 9:**
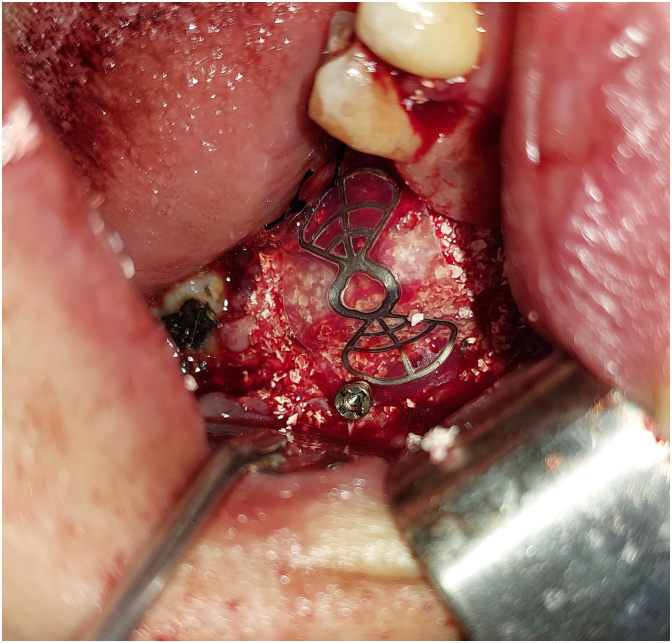
Fixing the PTFE membrane over the graft material.

The membrane was secured buccally using two fixing screws (InnoGenic™ GBR Kit) and extended lingually under the lingual flap (Fig. 9).

**Fig. 10:**
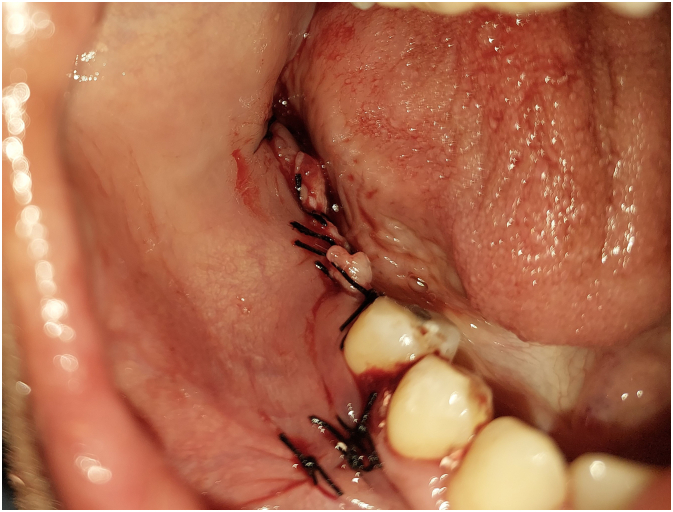
Flap closure.

Periosteal incisions were made in the buccal flap to achieve a tension-free primary closure. The flap was then closed using single interrupted sutures with 3/0 silk SURGIReal® (Fig. 10).

Medications prescribed included the previously mentioned antibiotic, along with an anti-inflammatory medication (600 mg ibuprofen twice daily for five days).

A 0.12 % chlorhexidine solution was applied three times a day until the sutures were removed to manage oral health. There was no wound dehiscence, no postoperative symptoms except for slight discomfort in the surgical area.

A cone beam computed tomography scan conducted six months after the surgery showed significant improvement in both vertical and horizontal bone dimensions. In addition, graft material showed a clear integration and density.

Upon exposure, the grafted site displayed a consistent color and texture, seamlessly blending with the surrounding native bone. Furthermore, the drilling process encountered consistent resistance, resulting in the production of dense, hard bone chips, providing strong evidence of successful bone graft integration.

Two dental implants (Mega gen ®) were placed following the release of a trapezoidal flap and the removal of the membrane and screws. The implants selected measured 3.5 mm in diameter and 13 mm in length.

## 4 Follow-up

**Fig. 11:**
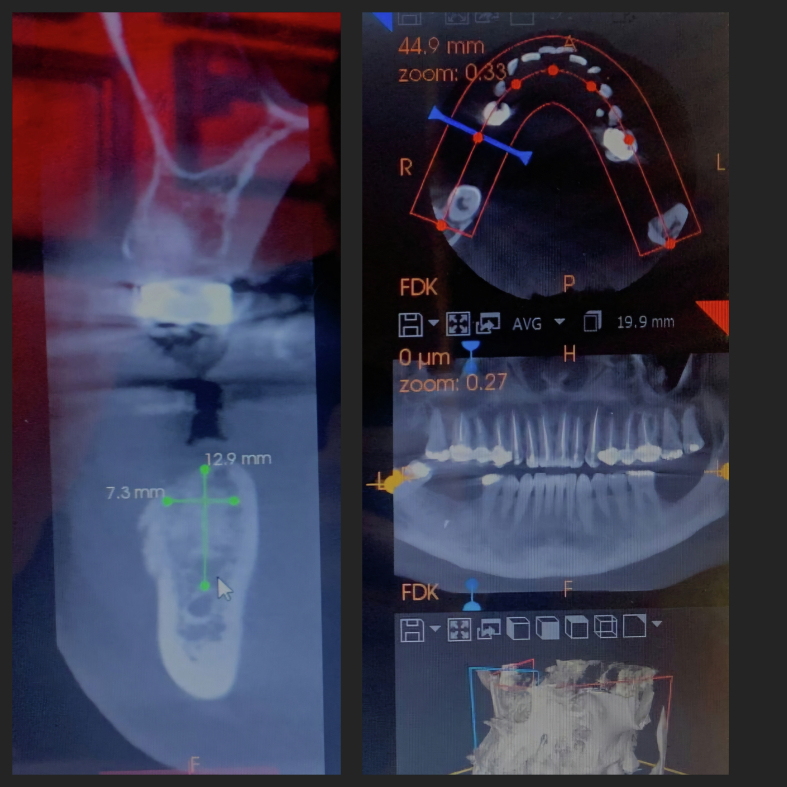
CBCT showing bone gain in vertical and horizontal dimensions in first molar area.

Cone Beam Computed Tomography (CBCT) revealed bone gain of nearly 7 mm in vertical dimension and about 8 mm in horizontal dimension in the first molar site (Fig. 11).

**Fig. 12:**
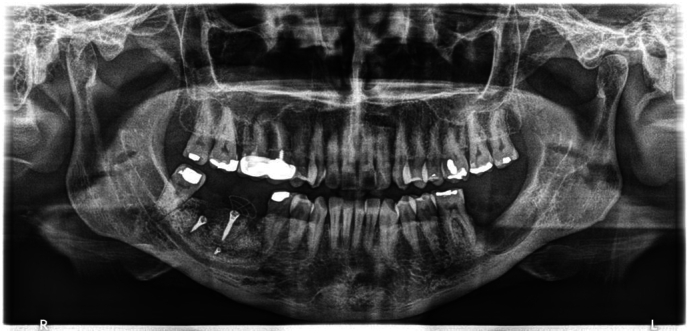
Panoramic x-ray follow-up.

**Fig. 13:**
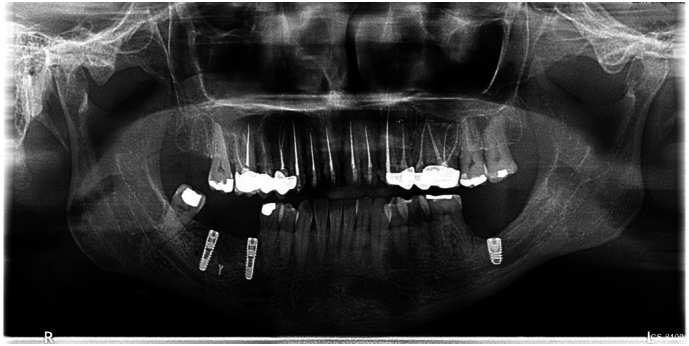
Dental implants placement.

A panoramic x-ray was conducted to check the insertion of the dental implants in the augmented site (Fig. 12, Fig. 13).

Three months later the implants were restored.

**Fig. 14:**
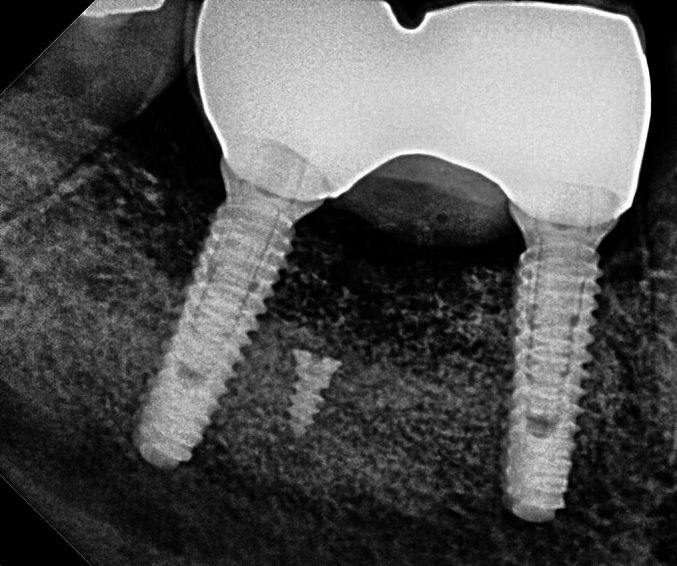
One year after implants loading.

A periapical x-ray was taken one year after loading to evaluate the long-term stability of the implants (Fig. 14).

## 5 Discussion

Guided bone regeneration in the posterior mandible presents significant challenges for oral surgeons. The rationale of GBR lies in effectively isolating non-osteogenic cells from the graft site using membranes to secure the bone graft [10].

Long-term studies have confirmed that vertically augmented bone, using GBR techniques, provides a safe and predictable base for implant placement, offering outcomes similar to those seen with native bone and minimizing potential complications [11].

Titanium-reinforced, non-resorbable PTFE membranes, due to their dimensional stability, provide more reliable support for bone augmentation compared to pliable, absorbable collagen membranes [6].

While space-making techniques like meshes and GBR membranes offer fewer complications than distraction osteogenesis or block grafting, flap dehiscence remains a significant concern. Furthermore, absorbable membranes have been linked to a higher incidence of complications compared to their non-resorbable counterparts. Therefore, meticulous flap closure without tension is critical to achieving optimal healing and higher success rates [12].

The tent pole technique offers a predictable and easily manipulated approach for horizontal ridge augmentation, minimizing complications [13].

Doan and Le demonstrated its effectiveness in increasing horizontal ridge dimension and facilitating implant placement.

This technique encourages bone growth and reduces graft resorption, likely due to the bone forming up to or even covering the screw heads [14].

Furthermore, the tent pole technique provides three-dimensional stabilization, distributing occlusal stresses evenly across the augmented area during healing. Thus, promoting graft integration and supporting bone graft against various stress patterns [7].

Autografting, the gold standard for bone repair, involves transplanting a section of the patient's own bone tissue to the defect site [15]. This approach provides all three formative bone properties, promoting revascularization and enhancing bone healing. To further promote revascularization, the use of decortication holes (creating multiple holes in the recipient site) and a combination of autograft and xenograft materials have shown promising results [16].

This clinical case demonstrated the surgical protocol for addressing severe bone loss in the posterior mandible with vertical and horizontal bone augmentation utilizing the titanium-reinforced PTFE membrane and the tent pole together to stabilize bone grafting procedure and reach significantly higher regeneration rates. Furthermore, utilizing guided bone regeneration with membranes only may collapse if not supported properly leading to less predictable outcomes [17].

The novelty of this case lies in the integration of the tent pole technique in the GBR procedure, which, in combination with the titanium-reinforced PTFE membrane, efficiently supported the grafted area, and enhanced the mechanical stability for the graft material. In addition, optimal space maintenance was achieved by using a tent screw to support the soft tissue and covering the graft with a Wi-Fi mesh to prevent soft tissue collapse and invasion. This combination of techniques led to more predictable outcomes in complex 3D bone augmentation. Thus, enabling dental implant insertion six months postoperatively.

Limitations of this study included a short follow-up period to fully assess the long-term effectiveness of this technique in achieving stable, high regeneration rates. Moreover, lack of histomorphometric analysis hindered a deeper understanding of the guided bone regeneration process.

The treatment protocol presented in this case report showed significant outcomes in three-dimensional bone regeneration, leading to more predictable results concerning restoring lost teeth with dental implants insertion.

## 6 Conclusion

As a cornerstone of oral surgery, alveolar bone grafting significantly impacts oral rehabilitation and patient well-being. This study proposes a potentially effective solution within the field of GBR, seeking to achieve durable and satisfying outcomes.

## Patient perspective

The patient recognized the significance of this surgical procedure in achieving favorable outcomes for oral rehabilitation and functional improvement.

## Consent

Written informed consent was obtained from the patient for publication of this study and accompanying images, a copy of the written consent is available for review by the Editor-in-Chief of International Journal of Surgery Case Reports.

## Ethical approval

Ethical approval was not required by the ethics committee of Tishreen University for this case report.

## Funding

The author declares that there is no source of funding.

## Author contribution

Dr. Nadim Sleman: Author of the manuscript including performing the surgical procedure, writing, and discussion.

## Guarantor

Dr. Nadim Sleman

## Research registration number

researchregistry10470

## Conflict of interest statement

The author declares that there is no conflict of interest.
